# Telomerase reverse transcriptase germline mutations and hepatocellular carcinoma in patients with nonalcoholic fatty liver disease

**DOI:** 10.1002/cam4.1078

**Published:** 2017-07-04

**Authors:** Benedetta Donati, Alessandro Pietrelli, Piero Pingitore, Paola Dongiovanni, Andrea Caddeo, Lucy Walker, Guido Baselli, Serena Pelusi, Chiara Rosso, Ester Vanni, Ann Daly, Rosellina Margherita Mancina, Antonio Grieco, Luca Miele, Stefania Grimaudo, Antonio Craxi, Salvatore Petta, Laura De Luca, Silvia Maier, Giorgio Soardo, Elisabetta Bugianesi, Fabio Colli, Renato Romagnoli, Quentin M. Anstee, Helen L. Reeves, Anna Ludovica Fracanzani, Silvia Fargion, Stefano Romeo, Luca Valenti

**Affiliations:** ^1^ Department of Pathophysiology and Transplantation Università degli Studi di Milano Fondazione IRCCS Ca’ Granda Ospedale Maggiore Policlinico Milan 20122 Italy; ^2^ Istituto Nazionale di Genetica Molecolare (INGM) Romeo ed Enrica Invernizzi Bioinformatics Group Milan 20122 Italy; ^3^ Sahlgrenska Center for Cardiovascular and Metabolic Research Wallenberg Laboratory Department of Molecular and Clinical Medicine University of Gothenburg Gothenburg SE‐405 30 Sweden; ^4^ Internal Medicine and Metabolic Diseases Fondazione IRCCS Ca’ Granda Ospedale Maggiore Policlinico Milan 20122 Italy; ^5^ The Freeman Hospital Newcastle upon Tyne Hospitals NHS Foundation Trust Newcastle upon Tyne NE7 7DN United Kingdom; ^6^ Division of Gastroenterology Department of Medical Sciences University of Torino Torino 10126 Italy; ^7^ Internal Medicine and Gastroenterology Area Fondazione Policlinico Universitario A. Gemelli Catholic University of Rome Rome 00168 Italy; ^8^ Section of Gastroenterology DIBIMIS University of Palermo Palermo 90127 Italy; ^9^ Clinic of Internal Medicine‐Liver Unit Department of Experimental and Clinical Medical Sciences University of Udine Udine 33100 Italy; ^10^ Department of Surgical Sciences Liver Transplantation Center University of Torino Torino 10126 Italy; ^11^ Liver Research Group Institute of Cellular Medicine Newcastle University Newcastle upon Tyne NE2 4HH United Kingdom; ^12^ Northern Institute for Cancer Research The Medical School Newcastle University Newcastle upon Tyne NE2 4HH United Kingdom; ^13^ Clinical Nutrition Unit Department of Medical and Surgical Sciences Magna Graecia University Catanzaro 88100 Italy

**Keywords:** Hepatocellular carcinoma, nonalcoholic fatty liver, rare germline mutations, telomerase reverse transcriptase, telomere

## Abstract

In an increasing proportion of cases, hepatocellular carcinoma (HCC) develops in patients with nonalcoholic fatty liver disease (NAFLD). Mutations in telomerase reverse transcriptase (hTERT) are associated with familial liver diseases. The aim of this study was to examine telomere length and germline *hTERT* mutations as associated with NAFLD‐HCC. In 40 patients with NAFLD‐HCC, 45 with NAFLD‐cirrhosis and 64 healthy controls, peripheral blood telomere length was evaluated by qRT‐PCR and *hTERT* coding regions and intron–exon boundaries sequenced. We further analyzed 78 patients affected by primary liver cancer (NAFLD‐PLC, 76 with HCC). Enrichment of rare coding mutations (allelic frequency <0.001) was evaluated by Burden test. Functional consequences were estimated in silico and by over‐expressing protein variants in HEK‐293 cells. We found that telomere length was reduced in individuals with NAFLD‐HCC versus those with cirrhosis (*P* = 0.048) and healthy controls (*P* = 0.0006), independently of age and sex. We detected an enrichment of *hTERT* mutations in NAFLD‐HCC, that was confirmed when we further considered a larger cohort of NAFLD‐PLC, and was more marked in female patients (*P* = 0.03). No mutations were found in cirrhosis and local controls, and only one in 503 healthy Europeans from the 1000 Genomes Project (allelic frequency = 0.025 vs. <0.001; *P* = 0.0005). Mutations with predicted functional impact, including the frameshift Glu113Argfs*79 and missense Glu668Asp, cosegregated with liver disease in two families. Three patients carried missense mutations (Ala67Val in homozygosity, Pro193Leu and His296Pro in heterozygosity) in the N‐terminal template‐binding domain (*P* = 0.037 for specific enrichment). Besides Glu668Asp, the Ala67Val variant resulted in reduced intracellular protein levels. In conclusion, we detected an association between shorter telomeres in peripheral blood and rare germline *hTERT* mutations and NAFLD‐HCC.

## Introduction

Following the epidemics of obesity and insulin resistance, nonalcoholic fatty liver disease (NAFLD) is becoming a major cause of hepatocellular carcinoma (HCC) in Western countries 1. NAFLD‐HCC patients are most commonly older males with type 2 diabetes and meeting criteria for at least one feature of the metabolic syndrome, mostly unaware of being affected by a progressive form of liver disease 2, 3, 4, 5. Although the growing rates of HCC may be due to the increased number of individuals with advanced fibrosis, NAFLD‐HCC develops in patients without cirrhosis more frequently than in liver disease caused by other factors, suggesting steatosis directly promotes hepatic carcinogenesis 5, 6, 7. This complex clinical presentation renders classic screening strategies for early detection of HCC ineffective.

Family history and genetic factors play important roles in the pathogenesis of progressive NAFLD and of HCC 8, 9. In particular, the common PNPLA3 I148M mutation increases the risk of NAFLD and favors hepatic carcinogenesis 1, 10, 11. However, although the presence of the 148M mutation has a relatively high specificity, it lacks sensitivity to be used as single marker for NAFLD‐HCC risk stratification 12. Therefore, there is still an urgent need of prognostic biomarkers able to identify NAFLD patients at risk of HCC.

Rare mutations inducing Mendelian diseases due to severe derangements in the function of encoded proteins may also be involved in predisposition to NAFLD‐HCC. Indeed, mutations in *APOB* have been linked to familial cases through predisposition toward development of severe steatosis 13. While acquired activating mutations in the telomerase reverse transcriptase (*hTERT*) promoter are commonly observed during hepatic carcinogenesis 14, loss‐of‐function germline mutations in *hTERT* can predispose to a spectrum of familial liver diseases characterized by steatosis 15 and possible evolution to cirrhosis and HCC 16, 17. We previously reported the occurrence of NAFLD‐HCC in a patient with an *hTERT* loss‐of‐function mutation 18. *TERT* encodes the catalytic reverse transcriptase subunit of the enzymatic complex responsible for maintaining telomere length. Telomeres are repetitive DNA sequences at the end of the genes responsible for protecting chromosomes tips, which shorten during each round of cell division 19. Therefore, telomere attrition is exacerbated in degenerative conditions characterized by chronic injury and regeneration with accelerated cell turnover 20. Shortened telomeres play a causal role in the pathogenesis of liver fibrosis by inducing senescence 21, 22, 23, 24, and eventually predispose to HCC because short telomeres do not effectively protect from chromosomal rearrangements during cell replication 25.

The aim of this study was to examine whether peripheral blood telomere length and in particular rare germline *hTERT* mutations (main outcome) are associated with NAFLD‐HCC development. Furthermore, we characterized the functional impact of the identified *hTERT* mutations by using both in silico and in vitro approaches.

## Materials and Methods

### Study design

The study design is shown in Figure S1. During phase 1 we investigated the presence of rare germline *hTERT* coding mutations. We defined rare coding mutations as variations with allelic frequency <0.001, according to two of largest frequency database available namely ExAC (Exome Aggregation Consortium) in the Non‐Finnish European (NFE) population and ESP (Exome Sequencing Project) European‐American (EA) population. The mutation frequency was compared to that of local controls and European individuals included in the 1000G (*n* = 503). Furthermore, we evaluated telomere length on a discovery cohort of Italian NAFLD‐HCC (*n* = 40), NAFLD‐cirrhosis (*n* = 45), and healthy individuals (*n* = 64) of comparable age and sex distribution. In phase 2, we attempted replication in a validation cohort including both Italian and Northern European subjects with NAFLD, who developed primary liver cancer (PLC, 97% with HCC). We subsequently conducted family studies to test for segregation of the *hTERT* mutations with liver disease. Finally, we examined the functional effects of the coding mutations identified by bioinformatics using a combination of in silico prediction tools and in vitro by cell studies with overexpression of recombinant wild‐type and mutated proteins.

### Patients

In phase 1, we enrolled 40 patients with NAFLD‐HCC: 20 from Policlinico Hospital of Milan, 4 from S. Giovanni Battista Hospital of Turin, 1 from the Gastroenterology Unit of Palermo, and the remaining 15 from the Hospital of Udine. The diagnosis of HCC was based on the EASL‐EORTC Clinical Practice Guidelines for management of hepatocellular carcinoma 26. Additionally, 45 patients affected by NAFLD cirrhosis were enrolled in order to confirm the association of the mutations eventually found with the carcinogenic phenotype (20 from Palermo and 25 from Milan). Secondary causes of steatosis were excluded on history, including alcohol abuse (≥ 30 g/day in M/F) and the use of drugs known to precipitate steatosis. Viral and autoimmune hepatitis, hereditary hemochromatosis, Wilson's disease, alpha‐1‐antitrypsin deficiency, and present or previous infection with HBV (HBsAg and HBsAb) and HCV were ruled out using standard clinical and laboratory evaluation as well as liver biopsy features. For all patients, complete clinical data and follow‐up are available in Table 1. Missing data (representing less than 5% for each category) have been replaced by the median for each category. Samples were collected from January 2012 until December 2013.

**Table 1:** Clinical features of subjects included in the study

	Discovery cohort				Validation cohort	
	Healthy (*n* = 64)	Cirrhosis (*n* = 45)	HCC (*n* = 40)	*P*	PLC (*n* = 78)	*P*
Age, years	59.1 ± 6.6	58.8 ± 8.7	66.3 ± 9.5	<0.0001	67.5 ± 8.4	<0.0001
Sex, F	19 (30)	15 (33)	13 (33)	0.606	11 (14)	0.0239
BMI	25.5 ± 2.6	30.5 ± 4.3	28.6 ± 4.0	0.0011	30.3 ± 5.4	<0.0001
T2DM, y	0	29 (64)	24 (60)	<0.0001	45 (58)	<0.0001
Fibrosis, F3‐4	0	45 (100)	32 (80)	<0.0001	61 (78)	<0.0001
Italian origin, y	64 (100)	45 (100)	40 (100)		50 (64)	
HCC, y	0	0	40 (100)		76 (97)	
*PNPLA3*, I148M				0.0086		<0.0001
I/I	36 (56)	9 (20)	15 (37)		16 (21)	
I/M	24 (38)	23 (51)	14 (35)		36 (46)	
M/M	4 (6)	13 (29)	11 (28)		26 (33)	

PLC, primary liver cancer; (), % values; y, yes; T2DM, type 2 diabetes mellitus; HCC, hepatocellular carcinoma; *P*,* P* value calculated as HCC versus healthy subjects.

We also analyzed a local ethnically matched control group of comparable sex distribution including 64 healthy blood donors without clinical and biochemical evidence of liver disease and no alcohol abuse 27. Presence of rare germline hTERT mutations was also searched for in 503 European individuals included in the 1000 Genomes database (http://www.internationalgenome.org).

In the second phase of the study, we examined a validation cohort collected after January 2014, including 78 patients affected by NAFLD‐PLC, 2 of whom had intrahepatic cholangiocarcinoma (28 from the Freeman Hospital of Newcastle upon Tyne, 24 from Turin, 25 from the Policlinico Gemelli of Rome and 1 from Milan). We did not exclude NAFLD‐associated intrahepatic cholangiocarcinoma in the validation cohort, since we could not rule out that germline mutations in hepatic stem cells might give rise to cancers with different phenotypes. All were of Caucasian ancestry. Clinical features are shown in Table 1.

Finally, in the family study we considered the relatives of two HCC patients belonging to the discovery cohort who carried hTERT mutations. In order to evaluate a genotype–phenotype correlation, liver enzymes were measured and abdominal ultrasonography and liver stiffness measurement were performed in family members of the probands.

The study protocol conformed to the ethical guidelines of the 1975 Declaration of Helsinki was approved by the ethical committee of the Fondazione IRCCS of Milan and was performed according to the recommendations of the hospitals involved. Informed consent was obtained from each patient or responsible guardian.

### Telomere length measurement

DNA was obtained from peripheral blood leukocytes or liver biopsies by phenol–chloroform extraction. Quality control was performed by evaluating 260/280 nmol/L absorbance ratio and by 1% agarose gel electrophoresis. Mean telomere length was measured by quantitative real‐time polymerase chain reaction (qPCR), as previously described 28, 29, 30, 31. Briefly, PCR was conducted in triplicate in a 7500 Fast Real Time PCR System (Life Technologies, Foster City, CA) and results are presented as ratio of telomere repeat copy number to 36B4 single gene copy number, calculated considering the relative quantity of the two distinct PCR products.

### *hTERT* sequencing

The prevalence of coding mutations in *hTERT* sequence was evaluated by Sanger sequencing on DNA previously extracted from peripheral blood leukocytes. The primers used for amplification and sequencing of the whole *hTERT* sequence, including the 16 exons and intron–exon boundaries, are listed in Table S1.

### Mutagenesis, cloning, and overexpression of *hTERT* variants

Wild‐type *hTERT* cDNA was synthesized and cloned in the pcDNA 3.1 vector with a V5 epitope tag at the C‐terminus by GeneArt Gene Synthesis (Thermo Fisher Scientific, Rockford, IL, USA). Single base‐pair changes resulting in the Ala67Val or Glu668Asp substitutions were introduced by overlap extension PCR cloning. Single base‐pair changes resulting into a proline to leucine (Pro193Leu or His296Pro) substitutions were introduced by site‐directed mutagenesis. A detailed protocol for mutagenesis is available upon request. The PCR products were cloned in pcDNA 3.1 vector (pcDNA 3.1 Directional TOPO expression kit; Invitrogen, Carlsbad, CA). The presence of the *hTERT* mutations and fidelity of each construct were confirmed by DNA sequencing. Human embryonic kidney cells (HEK‐293) were cultured in DMEM (Dulbecco's modified Eagle's medium) containing 10% FBS (fetal bovine serum). Expression plasmids (30 *μ*g/T‐75 flask) containing the human wild‐type hTERT or mutants were used to transfect HEK‐293 cells using Lipofectamine 3000 (Thermo Fisher Scientific) reagent according to the manufacturer's protocol. After 48 h cells were collected. Cells were lysed in M‐PER^®^ (Mammalian Protein Extraction Reagent, Thermo Fisher Scientific) containing complete protease inhibitors cocktail (Sigma‐Aldrich, Saint Louis, Missouri, USA) and analyzed by western blotting. The intensity of the western blotting bands was measured by Image Lab Software (Bio‐Rad) and expressed as arbitrary unit (AU). The highest value obtained was assigned as 1.

### Bioinformatics and statistical analyses

All the variants found in the sequencing experiment were functionally annotated using the *hTERT* RefSeq reference transcript NM_001193376. We defined rare coding mutations as variations not present in dbSNP (release 147) and 1000 Genomes Project (Phase 3) or described with a minor allele frequency (MAF) <0.001 according to ExAC NFE and ESP EA populations. The rare variants identified were submitted to the ClinVar database (www.ncbi.nlm.nih.gov/clinvar/; submission ID: SUB2041085 [MDI‐7607]).

For descriptive statistics, continuous variables are shown as mean and standard deviation, while categorical variables are presented as number and proportion. Telomere lengths comparisons have been conducted by generalized linear model corrected for sex and age.

Burden test was performed using the collapsing method CAST (Cohort Allelic Sum Test) available in the R package AssotesteR (http://CRAN.R-project.org/package=AssotesteR). Briefly, the genotype of the rare coding mutations for the individual of each group of patients and controls was summarized and collapsed into a single genetic score, taking into account both the number of mutated alleles in each group and the mutation frequency in controls. The association of this score with the trait was tested using Fisher's test. The association was considered statistically significant with *P* values lower than 0.05.

The impact of rare coding mutations on protein activity was predicted in silico by different bioinformatics algorithms including Polyphen‐2 (http://genetics.bwh.harvard.edu/pph2/), SIFT (http://sift.jcvi.org) and PROVEAN (http://provean.jcvi.org).

Statistical analyses were carried out using the JMP 12.0 statistical analysis software (SAS Institute, Cary, NC) and R statistical analysis software version 3.3.2 (http://www.R-project.org/). *P*s < 0.05 were considered statistically significant. The study methods and results have been reported according to the STROBE/STREGA guidelines for genetic association studies.

## Results

### Telomere length is reduced in peripheral blood leukocytes of NAFLD‐HCC patients

**Figure 1:**
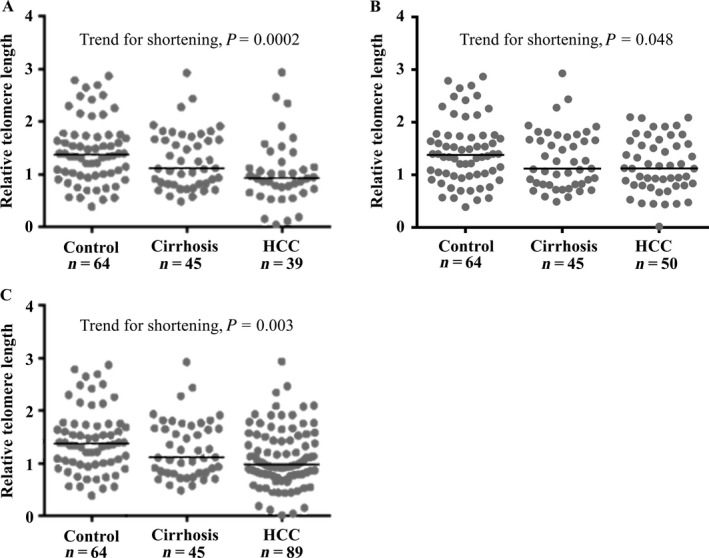
Telomere length is reduced in peripheral blood leukocytes of NAFLD‐HCC patients. Relative telomere length of patients included in the discovery cohort (A), in the validation cohort (B), and in the overall cohort (C) was reported in the figure. Overall cohort includes all the Italian NAFLD‐HCC patients (see Table 1). For the different comparison *P* values were calculated by linear generalized model corrected for sex and age considering log‐transformed data (Table S2). For all the specimens, telomere length was evaluated by qPCR as ratio of the relative quantity of the telomere PCR product and the reference gene (36B4).

Telomere length in peripheral blood leukocytes decreased with liver disease progression from healthy controls to NAFLD‐cirrhosis to NAFLD‐HCC (*P* = 0.005). The trend for shortening was maintained after adjusting the analysis for sex and age (*P* = 0.0002; Fig. 1A). In particular, telomere length was shorter in patients with HCC than in those with cirrhosis (median: 0.93 IQR: 0.66–1.24 vs. 1.12 IQR: 0.82–1.69; *P* = 0.014), and in healthy subjects (median: 1.38 IQR: 0.99–1.75; *P* = 0.0001). In an independent validation cohort of 50 Italian NAFLD‐HCC patients, we also detected a significant shortening of telomere length as compared to controls (NAFLD‐HCC validation cohort median: 1.12 IQR: 0.80‐1.60; *P* for trend=0.048; Fig. 1B). When we considered the overall cohort of NAFLD‐HCC samples evaluated (*n* = 89), the trend for shortening was again highly significant (*P* = 0.003), with NAFLD‐HCC patients having shorter telomeres as compared even to those with uncomplicated cirrhosis and to healthy individuals (NAFLD‐HCC overall cohort median: 0.98 IQR: 0.77–1.52; *P* = 0.048 and *P* = 0.0006, respectively; Fig. 1C).

### Rare *hTERT* coding mutations are enriched in NAFLD‐HCC patients

**Figure 2:**
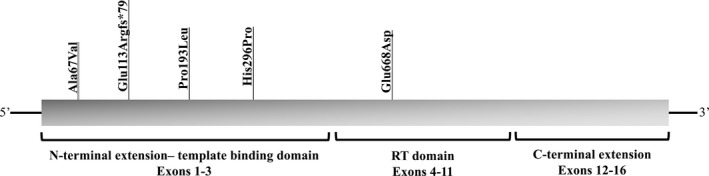
Rare coding mutations found in *hTERT* gene. Schematic representation of *hTERT* sequence showing the distribution of the rare coding mutations identified in the different domains.

The main study aim was to evaluate the prevalence of rare *hTERT* coding variants in NAFLD‐HCC patients. In the discovery cohort made up of Italian NAFLD‐HCC patients, we found four (three novel and one previously described) mutations. In particular, one HCC patient carried a frameshift mutation, Glu113Arg_fs*79, in the second exon of the sequence (Fig. 2 and Figure S2). Three NAFLD‐HCC patients carried missense mutations: Ala67Val, Pro193Leu, and Glu668Asp. The Ala67Val and Pro193Leu are both located in the N‐terminal (template‐binding domain). The Glu668Asp mutation is located in the catalytic domain (Fig. 2). All but the Ala67Val mutations were detected in heterozygosity. There was a significant enrichment in *hTERT* rare coding variants in NAFLD‐HCC patients as compared to local controls consistent of patients with NAFLD‐cirrhosis or healthy individuals, where no mutations were identified (mutated alleles prevalence 10%; *P* = 0.022 vs. cirrhosis, *P* = 0.008 vs. healthy subjects; *P* = 0.001 vs. controls overall). Burden test analysis confirmed the enrichment of rare *hTERT* coding mutations in NAFLD‐HCC (*P* = 0.020 vs. healthy controls; *P* = 0.045 vs. cirrhosis; Table 2). When we extended the control group to the 503 healthy European subjects from the 1000 Genomes Project database (http://www.internationalgenome.org/faq/can-i-get-phenotype-gender-and-family-relationship-information-samples), in which only one individual of Italian origin was a heterozygous carrier of a rare coding mutation in *hTERT*, we confirmed a strong enrichment of *hTERT* mutations in NAFLD‐HCC (*P* = 0.0001; Table 2).

**Table 2:** Description of rare nonsynonymous variations in *hTERT* gene found in subjects analyzed

Mutation	SNP_ID (dbSNP147)	Chr:Position (GRCh37)	Ref/Alt	European 1000 G (n.a. = 1006)	Discovery			Discovery RR (95% CI)	Discovery *P* value	Validation	Overall RR (95% CI)	Overall p value
					Healthy controls (n.a. = 128)	Cirrhosis (n.a. = 90)	HCC (n.a. = 80)			PLC (n.a. = 156)		
								76.6 (9.2–640.3)	0.005° 0.0001^		31.2 (3.8–256.5)	0.0005
Ala67Val^a^	–	5:1294905	G/A	–	–	–	2			–		
Glu113Argfs	–	5:1294664	G/GG	–	–	–	1			–		
Pro193Leu	rs751762765	5:1294423	G/A	–	–	–	1			–		
His296Pro	rs778187343	5:1294114	T/G	–	–	–	–			1		
Glu668Asp	–	5:1279532	C/G	–	–	–	1			–		
Val897Met	rs559028617	5:1264558	C/T	1	–	–	–			–		

The nonsynonymous variations found in subjects sequenced are listed in the table, together with the only mutation found with frequency <0.001 in “European 1000G.” All the mutations have been described with a frequency lower than <0.001 in both the ExAC NFE and the ESP EA. The table indicates the number of mutated alleles for each variation in the different groups analyzed and in the public database of “European 1000G.”

SNP, single nucleotide polymorphism; GRCh37, human genome assembly release 37; Ref/Alt, reference/alternative allele; PLC, primary liver cancer; n.a., number of alleles.

a Mutation in homozygosity; *P* values were calculated by Burden test considering HCCs versus controls (healthy subjects and cirrhosis; °) and versus European 1000G (^). Overall *P* value was calculated by Burden test considering overall HCCs versus controls (cirrhosis, healthy subjects, and European 1000G); RR, 95% CI (relative risk, 95% confidence intervals), was calculated for both discovery and overall cohorts versus all controls (cirrhosis, healthy subjects and European 1000G). Variants were annotated according to hg19/GRCh37, using the *hTERT* RefSeq reference transcript NM_001193376.

Interestingly, considering the distribution of known rare coding mutations in *hTERT* (see Telomere Database—http://telomerase.asu.edu), we observed an enrichment of variations in the N‐terminal of the gene (*P* = 0.037), as compared to catalytic and C‐terminal domains.

Besides rare variants, common missense mutations in *hTERT* were found in HCC and cirrhotic patients and healthy controls, at the expected frequencies (Ala279Thr, His412Tyr, and Ala1062Thr; Table S3).

### *hTERT* mutations in a European cohort of NAFLD‐PLC patients

We further sequenced *hTERT* in an independent cohort comprising 78 patients affected by PLC in NAFLD, 64% of Italian origin, 97% with HCC (Table 1). Here, we found one heterozygous carrier of a rare missense mutation (His296Pro; Fig. 2 and Figure S2) affected by intrahepatic cholangiocarcinoma. There was no significant enrichment in *hTERT* mutations between the validation cohort and controls (*P*=NS). However, when we considered the overall cohort of cancer patients (*n* = 118), as compared to all available controls (from discovery cohort and the 1000 Genomes database, *n* = 612), we confirmed a significant enrichment of rare germline *hTERT* variants in subjects affected by tumor developed in NAFLD (*P* = 0.0005; Table 2), which remained significant after the exclusion of the only two PLC patients without HCC (*P* = 0.003).

### Clinical features of patients carrying rare *hTERT* mutations

The clinical features of patients positive and negative for the presence of the rare *hTERT* mutations are shown in Table 3. We found that the prevalence of female sex was higher in carriers of *hTERT* mutations (*P* = 0.03). We did not observe any significant difference in the distribution of age, BMI, diabetes, and the presence of cirrhosis between the two groups (*P* = NS). The genetic risk factor PNPLA3 I148M was equally distributed between the two groups (*P* = 0.88). No significant difference was detected in peripheral blood telomere length between patients positive and those negative for *hTERT* mutations (not shown).

**Table 3:** Clinical features of 118 patients who developed primary liver cancer (PLC) in NAFLD stratified by carriage of rare *hTERT* mutations

*hTERT* mutation	PLC cohort overall		*P*
	Yes (*n* = 5)	No (*n* = 113)	
Age, years	70.4 ± 10.8	67.0 ± 8.8	0.52
Sex, F	3 (60)	21 (19)	0.03
BMI, kg/m^2^	28.8 ± 5.0	29.9 ± 5.3	0.65
T2DM, y	3 (60)	66 (58)	0.94
Fibrosis, F3‐4	4 (80)	89 (79)	0.93
PNPLA3, I148M			0.90
I/I	1 (20)	30 (27)	
I/M	2 (40)	48 (42)	
M/M	2 (40)	35 (31)	
HCC/CC	4/1 (75/25)	112/1 (99/1)	0.001

PLC, primary liver cancer; (), % values; y, yes; T2DM, type 2 diabetes mellitus; *P*,* P* value calculated as patients carriers of *hTERT* mutations versus not carriers; HCC, hepatocellular carcinoma; CC, cholangiocarcinoma.

### Family study

To investigate the pathogenicity of the mutations identified, we examined whether these cosegregated with liver disease or other pathological phenotypes. We were able to genotype and phenotype some relatives of the probands carrying the Glu668Asp (Family A, Figure S3) and Glu113Argfs (Family B, Figure S3) mutations. In Family A, one son of the proband carried the mutation in heterozygosity and already showed traits of liver damage (NAFLD and increased liver enzymes) despite a relatively young age. Moreover, the mother and the sister of the proband were affected by a phenotype very likely related to telomere disease (cryptogenic cirrhosis and idiopathic pulmonary fibrosis, respectively), but they were already deceased at the time of the referral and could not be evaluated. In Family B, the proband's brother, who also carries the Glu113Argfs mutation, has altered liver function tests. One of the younger daughters and a nephew of the proband are also heterozygous carriers of the mutation but with no phenotypic signs, possibly due to the young age.

### Functional evaluation of *hTERT* mutations

In order to investigate whether the mutations found could affect the catalytic activity of hTERT, their effect was modeled in silico using predictive bioinformatics algorithms. In addition to the Glu113Argfs frameshift mutation, which causes a premature termination of protein synthesis and is frankly damaging, the Glu668Asp variant was also predicted to be deleterious by two out of three prediction tools (Table S4). Conversely, the amino acid substitutions Ala67Val, Pro193Leu, and His296Pro were not predicted to disrupt the activity of the protein (Table S4), which does not rule out that they may impact on the DNA binding of TERT.

**Figure 3:**
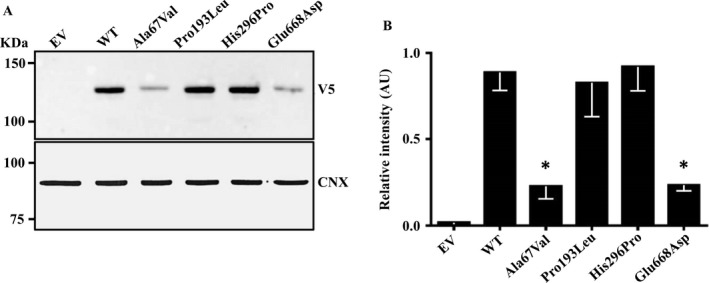
The hTERT Ala67Val and Glu668Asp variants result in a reduction of the intracellular protein levels. Effect of missense mutations on hTERT protein levels in HEK‐293 cells (A). Human TERT was transiently overexpressed in HEK‐293 cells. HEK‐293 cells were transfected with hTERT wild‐type and mutant forms cloned in pcDNA 3.1 vector for 48 h; recombinant hTERT protein levels were examined on cell lysate by western blotting analysis by using a V5 antibody. Empty vector was used as negative and calnexin was used as loading control. Quantification of western blotting bands (B). The graph bar represents intracellular protein levels expressed as mean and standard deviation (whisker) of three independent experiments. Protein levels were quantified by Image Lab Software (Bio‐Rad).

To gain further insight into the consequences of *hTERT* mutations, we transiently overexpressed the wild‐type and the missense mutants in human HEK‐293 cells. We observed a substantial reduction in the recombinant intracellular protein synthesis of the Ala67Val and Glu668Asp mutations as compared to the wild‐type protein (Fig. 3). There was virtually no change in the protein synthesis of the Pro193Leu and His296Pro mutants as compared to the wild‐type protein (Figure 3).

## Discussion

NAFLD‐HCC is an increasingly common cause of liver‐related mortality in Western countries, due to rising incidence and lack of adequate biomarkers allowing early diagnosis 1. NAFLD and HCC have a heritable component, but the genetic determinants influencing onset and progression of HCC remain mostly unknown, with rare genetic mutations possibly playing a role in triggering disease development 8, 9, 13. In this study, we examined whether mutations in *hTERT*, a key player in hepatic fibrosis progression and carcinogenesis 14, 17, 21, 32, are associated with NAFLD‐HCC development.

Consistently with literature data obtained in other liver diseases 25, we observed a progressive shortening of peripheral telomere length, from healthy controls to patients with NAFLD‐cirrhosis and finally to those who developed HCC. This trend for shortening was confirmed in two independent groups of NAFLD‐HCC patients as compared to a local cohort of patients with cirrhosis and healthy individuals, but the lack of an independent cohort of patients with NAFLD‐cirrhosis at high risk of HCC represents a limitation of this study. In keeping with these observations, previous studies demonstrated that telomere attrition is involved in the progression of liver disease, as well as in other chronic degenerative conditions 21, 22, 33, 34. Indeed, shorter telomeres most likely lead to exhaustion of tissue staminal compartments, senescence, and fibrosis, which may involve the lung, liver, and blood, and predispose to a wide spectrum of cancers by favoring genomic instability 35. Not only genetic alterations in telomerase complex genes, but also environmental risk factors for degenerative diseases, such as smoke and pollution, have been associated with peripheral blood telomere shortening, reflecting whole body exposure 34, 36. It is therefore possible that shorter telomeres may reflect both genetic predisposition and environmental factors exposure in NAFLD‐HCC patients (Fig. S4). However, it should be specified that our data indicate that despite it is associated with NAFLD‐HCC, shorter telomere length of peripheral blood leukocytes lacks sufficient diagnostic accuracy to be considered a novel biomarker to stratify HCC risk.

The main study hypothesis was that *hTERT* germline mutations favor the development of HCC in NAFLD. We focused on rare germline mutations determining an alteration of protein sequence because these are more likely to alter protein function 37, 38. Consistently, in the NAFLD‐HCC discovery cohort, we observed a strong enrichment in rare and novel *hTERT* mutations, more than 76‐fold as compared to the frequency of analogous mutations in local controls and healthy individuals of matched ethnicity in public databases. In further analysis, we considered as population controls only apparently healthy Europeans individuals included in the 1000 Genomes database. These data are consistent with the hypothesis that rare *hTERT* mutations predispose to NAFLD‐HCC.

The frequency of *hTERT* mutations was lower in a larger European validation cohort, but among NAFLD‐PLC patients (most of them affected by HCC) overall there still was a significant 31‐fold enrichment in *hTERT* mutations. The higher prevalence of mutations in the discovery cohort may possibly be ascribed to a higher proportion of individuals without strong cofactors for progressive NAFLD such as obesity and male sex, so that genetic factors may have played a larger role. Interestingly, despite HCC more frequently developing in males, we observed a higher prevalence of *hTERT* mutations in female patients with NAFLD‐PLC, so that the lower proportion of females in the validation cohort may have reduced the probability of finding mutations carriers. As one mutation carrier was actually diagnosed with intrahepatic cholangiocarcinoma, we cannot exclude the possibility that *hTERT* genetic variations represent a pathogenic risk factor also for cholangiocarcinoma, but conclusions could not be drawn based on a single case. Despite we have studied the largest cohort of NAFLD‐HCC patients with genetic characterization so far reported, collaborative prospective studies are needed in order to confirm this association in a larger cohort, as well as in non‐European individuals.

On the other hand, common missense variants in hTERT, including Ala279Thr, His412Tyr, and Ala1062Thr, previously reported to confer increased risk of telomere disease 39, 40, 41, were not differently represented between HCC cases and healthy controls. Even if these genetic variants may possibly confer subtle alterations in hTERT activity, we can reasonably exclude that they have a strong impact on telomere dysfunction; otherwise they would have undergone a strong negative selection (purification) during evolution. Our study was not sufficiently powered to detect a moderate effect on the risk of progressive NAFLD.

Besides the fact that are rare or novel mutations, other clues suggest a role for the identified genetic variants in the pathogenesis of NAFLD‐HCC. The first one is that mutations tended to cosegregate within the N‐terminal domain of *hTERT*, which is involved in telomere binding. Indeed, it is know that mutations in specific *hTERT* domains tend to determine specific pathological phenotypes 35. Second, in two families mutations cosegregated with liver disease and other phenotypes shared by telomere diseases in older individuals. As telomeropathies are age‐dependent degenerative conditions, a longer follow‐up will be necessary to better characterize the penetrance of these genetic variants in younger carriers. Furthermore, in silico analysis predicted functional consequences for Glu113Arg_fs, determining an early termination of protein and the Glu668Asp mutation in the catalytic domain. However, the ability of bioinformatics tools to predict the interaction of the other N‐terminal domain mutations with telomeres and interacting proteins is quite limited, as they are built to predict how an amino acid substitution influence protein activity.

To gain further insight into the functional consequences of *hTERT* mutations, we transiently overexpressed the wild‐type and the missense variants. We did not test in vitro the Glu113Argfs*79 mutation because of the high likelihood to undergo mRNA decay due to the severe damage caused by the frameshift 42. We observed that the Ala67Val and the Glu668Asp mutations resulted in a severe reduction in the intracellular protein levels as compared to the wild type, suggesting that the Ala67Val, carried in homozygosity by one NAFLD‐HCC patient, may induce a reduction in telomerase expression due to altered protein translation or stability. On the other hand, the remaining two mutations detected in NAFLD‐HCC patients without a clear pathogenic effect: the Pro193Leu and His296Pro mutations in the N‐terminal template‐binding domain, that do not likely influence the catalytic activity of hTERT, but may result in a reduction of the binding ability of hTERT, inducing novel telomerase functions in tumorigenesis independently of hTERC 43. For example, hTERT can act as a transcription factor in the Wnt‐*β*‐catenin signaling pathway, regulating the expression of procarcinogenic Wnt target genes 44. As the bioinformatics and in vitro approaches were not suitable to test these hypotheses and to evaluate the effective impact of these mutations on the different functions of telomerase, further studies are required to better investigate their possible causal role and mechanism in determining HCC predisposition.

As many other proteins are included in telomerase complex and participate to telomere elongation, we cannot exclude that mutations in other genes involved in telomere regulation play a role in telomere attrition in NAFLD‐HCC. Indeed, telomere length in peripheral blood was shortened irrespective of the presence of *hTERT* mutations in NAFLD‐HCC patients. Additional genetic factors should be investigated by next‐generation sequencing in order to examine whether carriage of rare genetic risk variants may account for apparently sporadic cases of NAFLD‐HCC. In fact, our approach suggests that rare germline mutations altering the sequence of protein known to be involved in the pathogenesis of NAFLD and chronic liver disease may play an important role in NAFLD‐HCC predisposition, representing useful biomarkers for risk stratification, particularly in family members of affected individuals.

In conclusion, we detected an association between shorter peripheral blood telomeres and NAFLD‐HCC development, and found that rare germline mutations in *hTERT* seems predispose to NAFLD progression to HCC, potentially assisting the identification of high‐risk individuals that may warrant closer surveillance and may be included in chemopreventive trials.

## Conflict of interest

The authors have no conflict of interest to declare.

## Supporting information


**Table S1**. Primers used for PCR and Sanger sequencing in *hTERT* sequencing analysis.
**Table S2**. Values of significance (Fisher *t*‐test) relative to telomere length comparisons in discovery and validation cohorts.
**Table S3.** Description of common nonsynonymous variations in *hTERT* gene found in subjects analyzed. The nonsynonymous variations found in subjects sequenced with a frequency higher than 0.1% in both the ExAC NFE and the ESP EA are listed in the table. The table indicates the number of mutated alleles for each variation in the different groups and in the public database of “European 1000G.”
**Table S4.** In silico prediction of functional impact of *hTERT* mutations according to bioinformatics algorithms.
**Figure S1**. Study design
**Figure S2**. Electropherograms related to the identified rare coding mutations. The figure shows in the upper panel the mutated sequences, while the corresponding normals are represented in the panel below.
**Figure S3**. Family study. Family trees of patients carriers of Glu668Asp and Glu113Argfs mutations (Family A and Family B, respectively).
**Figure S4.** Chronic conditions associated with telomere shortening in peripheral blood. Telomere attrition may reflect both genetic predisposition and environmental factors exposure.

## EPIDEMIC Study Group Investigators

Guido Baselli, Benedetta Donati, Paola Dongiovanni, Silvia Fargion, Anna Ludovica Fracanzani, Serena Pelusi, Alessandro Pietrelli, Luca Valenti (Milano, Italy); Laura De Luca, Silvia Maier, Giorgio Soardo, (Udine, Italy); Elisabetta Bugianesi, Fabio Colli, Renato Romagnoli, Chiara Rosso, Ester Vanni (Torino, Italy); Antonio Craxi, Stefania Grimaudo, Salvatore Petta (Palermo, Italy); Antonio Grieco, Luca Miele (Roma, Italy); Quentin M. Anstee, Ann Daly (Newcastle upon Tyne, UK); Andrea Caddeo, Rosellina Margherita Mancina, Piero Pingitore, Stefano Romeo (Gothenburg, Sweden).
