# Supplementary material for: Telomerase reverse transcriptase germline mutations and hepatocellular carcinoma in patients with nonalcoholic fatty liver disease
Source: Cancer Med. 2017 Jul 4;6(8):1930–40. doi: 10.1002/cam4.1078 (PMC5548883; doi:10.1002/cam4.1078)
Supplement: Supplementary file 1 — Table S1. Primers used for PCR and Sanger sequencing in hTERT sequencing analysis. Table S2. Values of significance (Fisher t‐test) relative to telomere length comparisons in discovery and validation cohorts. Table S3. Description of common nonsynonymous variations in hTERT gene found in subjects analyzed. The nonsynonymous variations found in subjects sequenced with a frequency higher than 0.1% in both the ExAC NFE and the ESP EA are listed in the table. The table indicates the number of mutated alleles for each variation in the different groups and in the public database of “European 1000G.” Table S4. In silico prediction of functional impact of hTERT mutations according to bioinformatics algorithms. Figure S1. Study design Figure S2. Electropherograms related to the identified rare coding mutations. The figure shows in the upper panel the mutated sequences, while the corresponding normals are represented in the panel below. Figure S3. Family study. Family trees of patients carriers of Glu668Asp and Glu113Argfs mutations (Family A and Family B, respectively). Figure S4. Chronic conditions associated with telomere shortening in peripheral blood. Telomere attrition may reflect both genetic predisposition and environmental factors exposure. [file CAM4-6-1930-s001.doc]

**Table S1.** **Primers used for PCR and Sanger sequencing in *hTERT* sequencing analysis.**

| **Exon** | **Sequence 5’ 3’** |
| --- | --- |
| 1-2 Forward | GAGTTTCAGGCAGCGCTGCGT |
| 1-2 Reverse | CTTGTCGCCTGAGGAGTAGAG |
| 1-2 Seq Forward* | CAACACGGTGACCGACGCACT |
| 1-2 Seq Reverse* | CAGGTGAACCAGCACGTCGTC |
| 2 Forward | CAGGACGCGTGGACCGAGTGA |
| 2 Reverse | GTGAACCTCGTAAGTTTATGC |
| 3 Forward | GTGATCTGGATGTGGCATGT |
| 3 Reverse | GGTGTTCCAGGACTTCGAGA |
| 4 Forward | GTCTGTTGTCTGGCTGAGCA |
| 4 Reverse | GCTCAAACGCACTTCTGTTTA |
| 5 Forward | ACTTGGCCGGATCCACTT |
| 5 Reverse | CACTCCCAAGGTCCAGCA |
| 6 Forward | CGTGGCCACTGTCAGTCT |
| 6 Reverse | CAGAGACACACATCCTGGACA |
| 7 Forward | CCACATTTGTGGCTCATGC |
| 7 Reverse | TCATGAGCCCAGTGATTGC |
| 8 Forward | GTCCTGCCTGTCTCAGCAC |
| 8 Reverse | GAAGGGGCAGGAGAGAGGT |
| 9 Forward | AGTGTACGCATGTCCAGCAC |
| 9 Reverse | AATCAACCCCCACCCAAG |
| 10 Forward | CCGCTTTGGAGAATGTTACTT |
| 10 Reverse | TGGAGGTCCCCACAGACA |
| 11 Forward | TCAGGTTACCTCCTGGGTGA |
| 11 Reverse | CCACACGGAAGCAGAGGT |
| 12 Forward | GCAGGAGGCTCTTTGGAG |
| 12 Reverse | TCCTGAACTCTGAACTCTGTG |
| 13 Forward | CCAGAGAGGTTTCTACCGTTT |
| 13 Reverse | GGTCAGAGGTGAGCAGAGC |
| 14 Forward | ACGAGCACCGTCTGATTAGG |
| 14 Reverse | AGGCACTGCTGCCACTGA |
| 15 Forward | CAGCTTTCCGGTGTCTCCT |
| 15 Reverse | GGGCGTTCAAGGATGACC |
| 16 Forward | CACCTCTGGCCTCTTCTGG |
| 16 Reverse | GACAGGGCTGCTGGTGTCT |

*Primer used only for sequencing

**Table S2.** **Values of significance (Fisher T-test) relative to telomere length comparisons in discovery and validation cohorts.**

|  | P value | | |
| --- | --- | --- | --- |
|  | Discovery | Validation | Overall |
| HCC vs Healthy | 0.0001 | 0.008 | 0.0006 |
| HCC vs Cirrhosis | 0.0144 | 0.16 | 0.048 |
| HCC vs no HCC | <0.0001 | 0.041 | 0.0003 |
| Cirrhosis vs Healthy | 0.2364 | - | - |
| Liver disease vs Healthy | 0.0067 | 0.022 | 0.0049 |

**Table S3.** **Description of common non-synonymous variations in *hTERT* gene found in subjects analyzed.** The non-synonymous variations found in subjects sequenced with a frequency higher than 0.1% in both the ExAC NFE and the ESP EA are listed in the table. The table indicates the number of mutated alleles for each variation in the different groups and in the public database of “European 1000G”.

|  |  | |  |  |  | Discovery | | |  | Validation |  |
| --- | --- | --- | --- | --- | --- | --- | --- | --- | --- | --- | --- |
| Mutation | SNP_ID  (dbSNP147) | Chr:Position  (GRCh37) | | Ref/Alt | European 1000G (n.a.=1006) | Healthy controls  (n.a.=128) | Cirrhosis  (n.a.=90) | HCC  (n.a.=80) | Discovery  p value | PLC  (n.a.=156) | Overall p value |
| Ala279Thr | rs61748181 | 5:1294166 | | C/T | 36 | 4 | 3 | 2 | 0.613*  0.751^§^ | 6 | 0.925 |
| His412Tyr | rs34094720 | 5:1294166 | | C/T | 3 | 1 | - | - | 0.625*  0.544^§^ | - | 0.842 |
| Ala1062Thr | rs35719940 | 5:1254594 | | G/A | 22 | 1 | - | - | 0.544*  0.518^§^ | 3 | 0.518 |

SNP (single nucleotide polymorphism); GRCh37 (human genome assembly release 37); Ref/Alt (Reference/Alternative allele); PLC (primary liver cancer); n.a. (number of alleles); p values were calculated by Chi Square test considering HCCs vs Controls (healthy subjects and cirrhosis; ^§^) and vs European 1000G (*); Overall p value was calculated by Chi Square test considering overall HCCs vs Controls (cirrhosis, healthy subjects and European 1000G). Variants were annotated according to hg19/GRCh37, using the *hTERT* RefSeq reference transcript NM_001193376.

**Table S4. *In silico* prediction of functional impact of *hTERT* mutations according to bioinformatic algorithms.**

|  | **hTERT *variants*** | | | | |
| --- | --- | --- | --- | --- | --- |
|  | Ala67Val | Glu113Argfs | Pro193Leu | His296Pro | Glu668Asp |
| **Polyphen2** | Benign | Damaging | Benign | Benign | Probably damaging |
| ***score*** | 0.011 |  | 0.000 | 0.160 | 1.000 |
| **SIFT** | Tolerated | Damaging | Tolerated | Tolerated | Damaging |
| ***score*** | 0.16 |  | 0.38 | 0.21 | 0.04 |
| **Provean** | Neutral | Deleterious | Neutral | Neutral | Neutral |
| ***score*** | -1.210 |  | -1.08 | -1.025 | -1.724 |

**Figure S1. Study design**

**
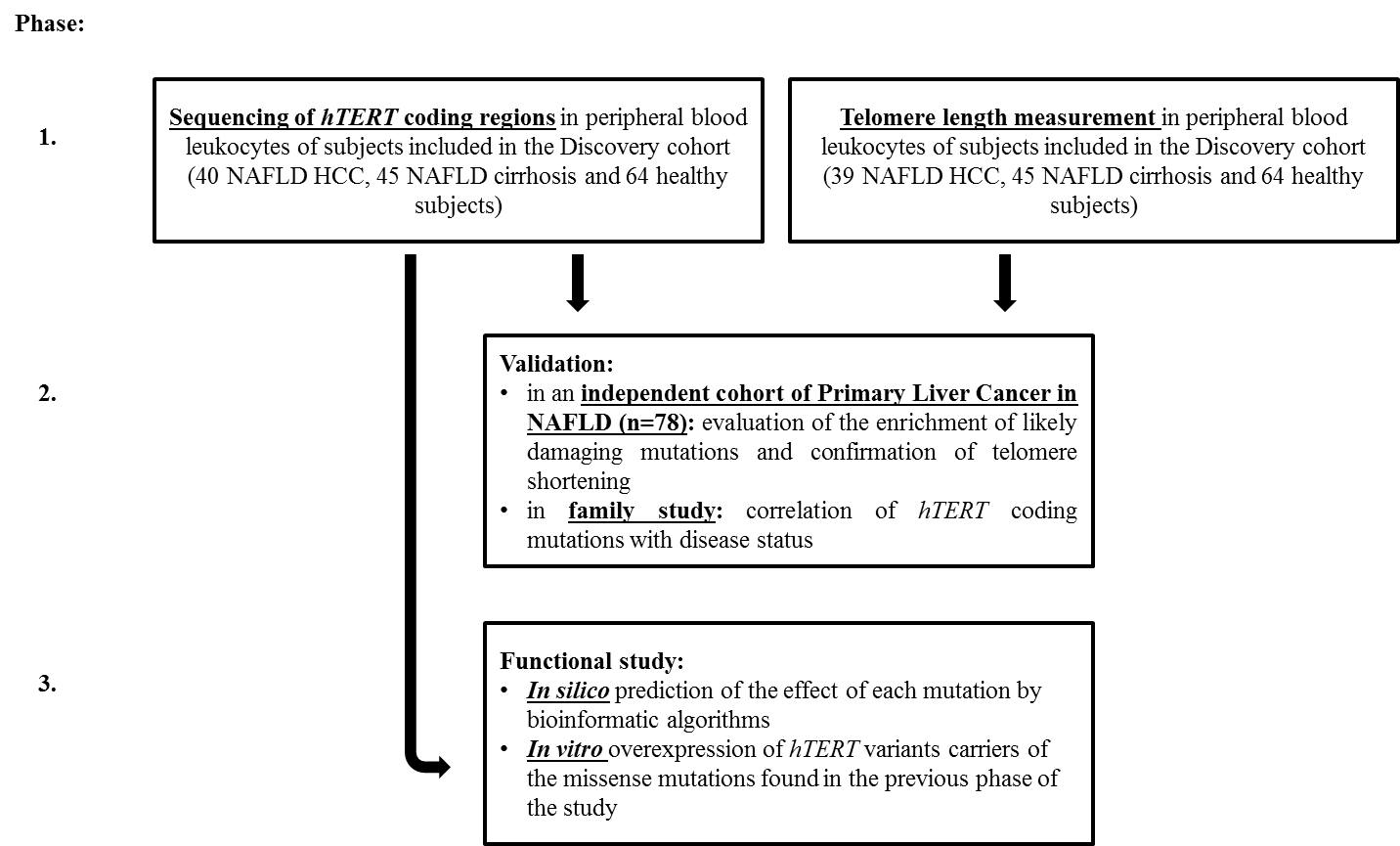
**

**Figure S2. Electropherograms related to the identified rare coding mutations.** The figure shows in the upper panel the mutated sequences, while the corresponding normals are represented in the panel below.


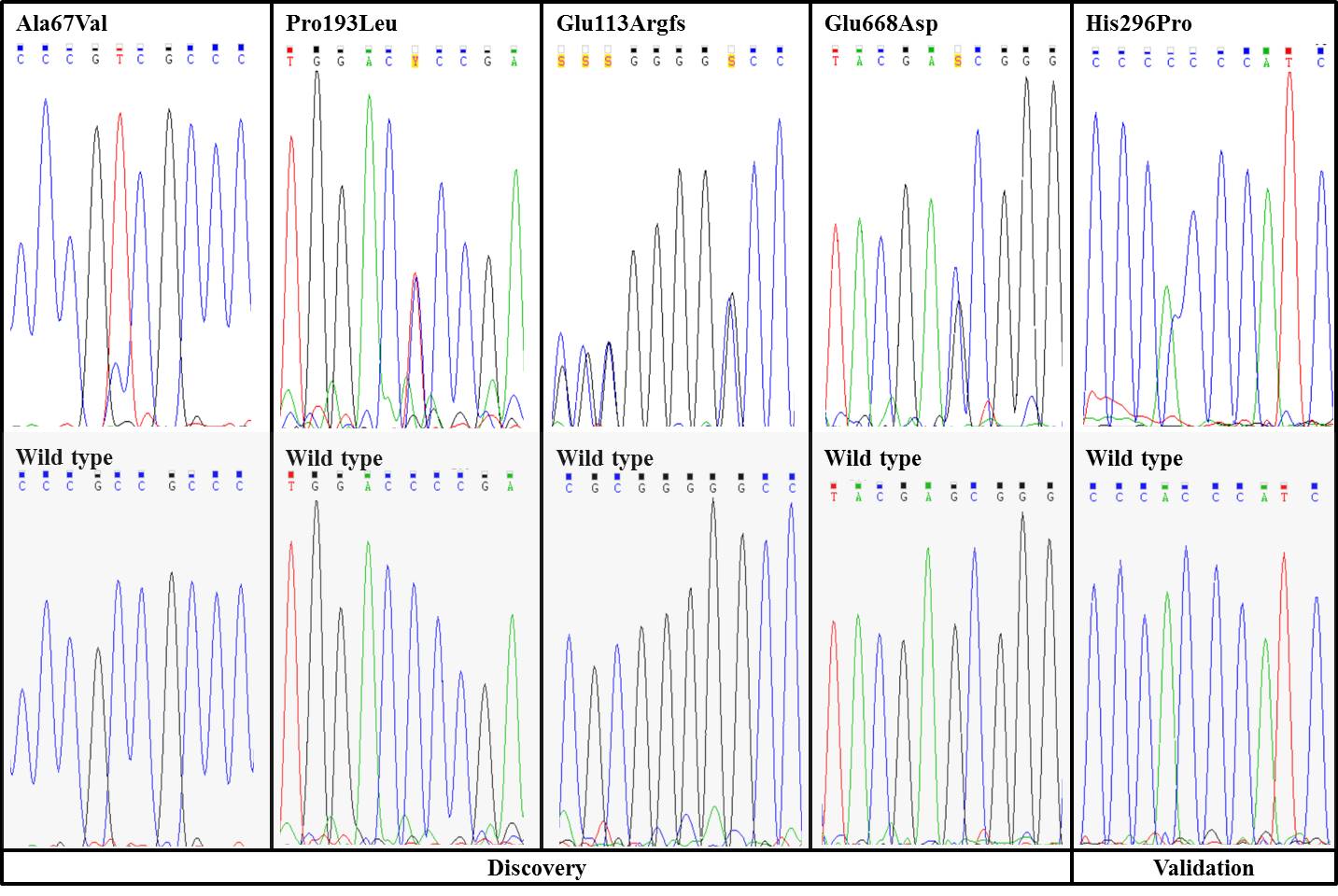


**Figure S3. Family study.** Family trees of patients carriers of Glu668Asp and Glu113Argfs mutations (Family A and Family B respectively).

**
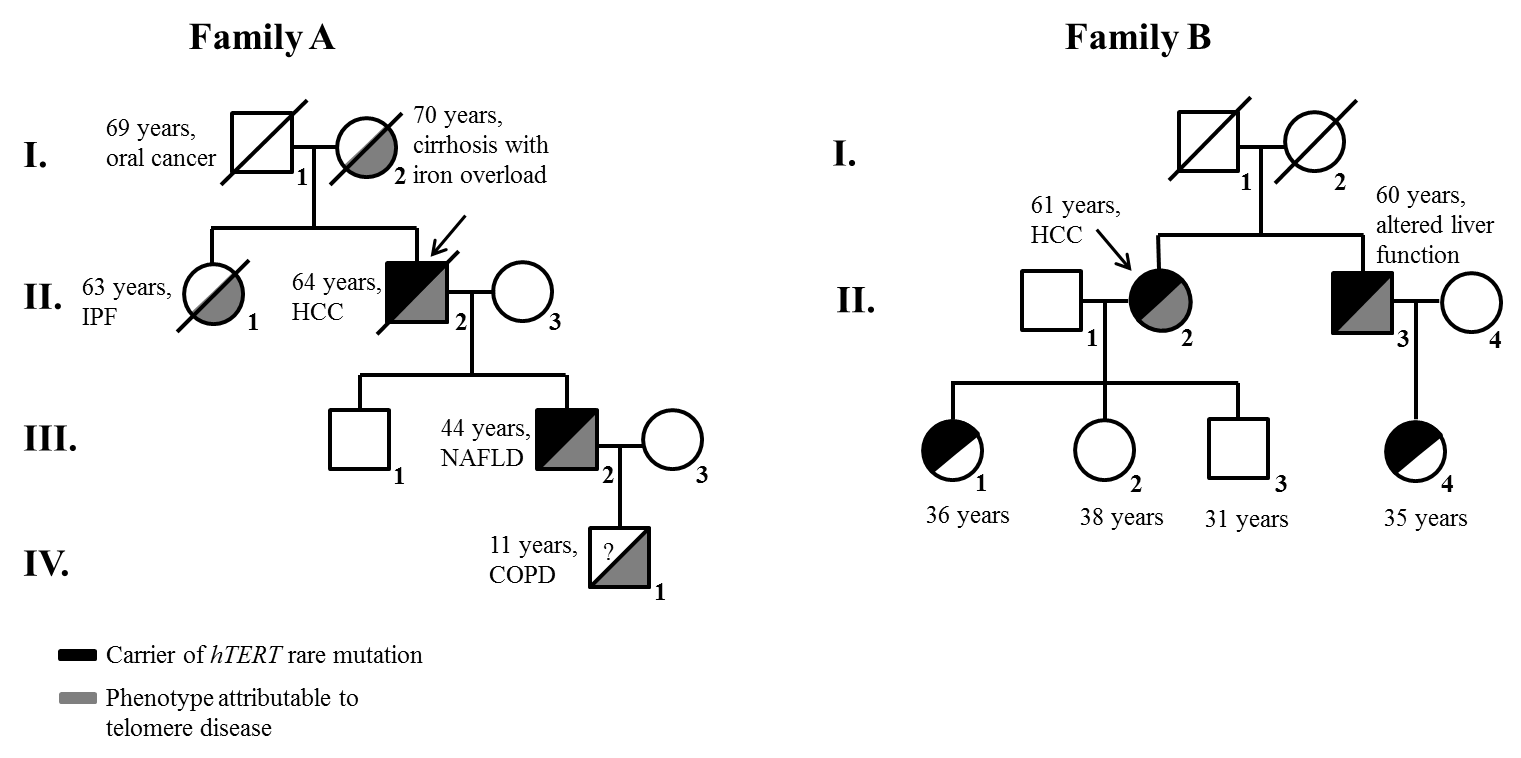
**

**Figure S4. Chronic conditions associated with telomere shortening in peripheral blood.** Telomere attrition may reflect both genetic predisposition and environmental factors exposure.

**
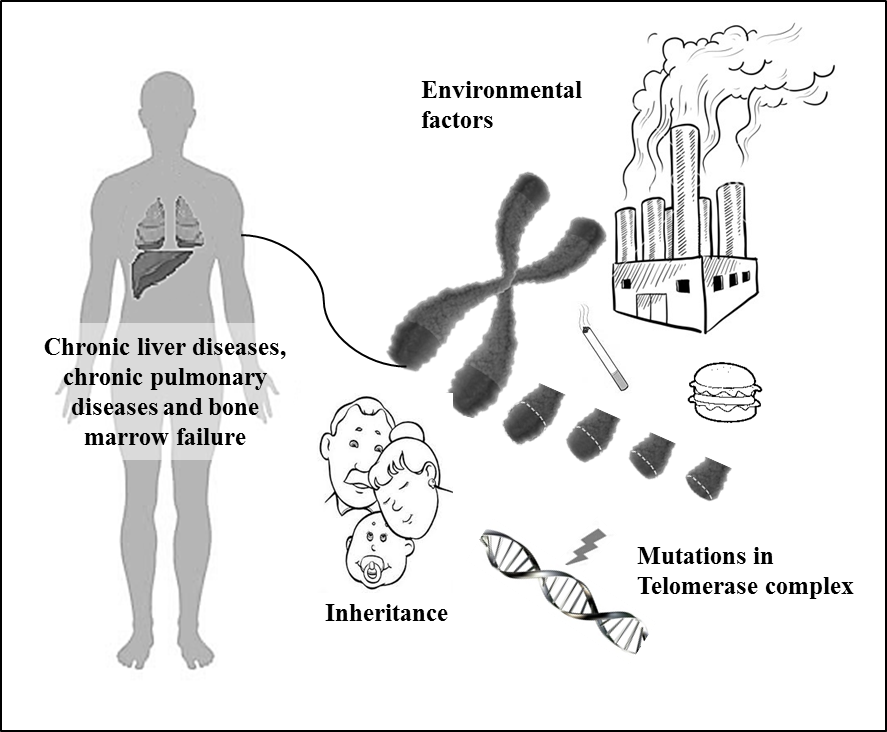
**
